# Valvular Heart Disease and the Importance of Inspiratory Muscle Training in Cardiovascular Rehabilitation

**DOI:** 10.7759/cureus.119102

**Published:** 2026-11-04

**Authors:** Bruno Bordoni, Bruno Morabito, Luigi Pianese, Gianmarco Cannadoro, Allan R Escher

**Affiliations:** 1 Physical Medicine and Rehabilitation, Foundation Don Carlo Gnocchi, Milan, ITA; 2 Cardiovascular Medicine, Associazione Italiana di Riabilitazione Cardiovascolare, Milan, ITA; 3 Physical Medicine and Rehabilitation, School of Osteopathic Centre for Research and Studies, Milan, ITA; 4 Cardiovascular Medicine, Associazione Italiana di Riabilitazione Cardiovascolare, Rome, ITA; 5 Physical Medicine and Rehabilitation, Research, Rome, ITA; 6 Cardiopneumology, Foundation Don Carlo Gnocchi, Milan, ITA; 7 Oncologic Sciences, University of South Florida Morsani College of Medicine, Tampa, USA; 8 Anesthesiology/Pain Medicine, H. Lee Moffitt Cancer Center and Research Institute, Tampa, USA

**Keywords:** airc, aortic valve, cardiac rehabilitation, mitral valve, pulmonary valve, tricuspid valve, valvular heart disease

## Abstract

Valvular heart disease (VHD) is a major cause of global cardiovascular morbidity and mortality. Treatment is either surgical or transcatheter, as there is currently no conservative pharmacological approach. Cardiovascular rehabilitation (CR) is indicated before or after invasive medical interventions to facilitate the patient's functional recovery. While CR is common practice for many cardiovascular conditions, guidelines specifically addressing VHD are lacking. Another gap concerns the use of inspiratory muscle training (IMT).

This narrative review discusses the importance of routinely incorporating IMT into CR programmes, offering hypotheses and recommendations for setting IMT parameters from the perspective of the Italian Association for Cardiovascular Rehabilitation (AIRC). Further research is needed to improve our understanding of rehabilitation and VHD.

## Introduction and background

Valvular heart disease (VHD) is a major cause of global cardiovascular morbidity and mortality [1]. VHD can be classified as acquired (developing over the course of a lifetime) or congenital (present in childhood), with dysfunction manifesting as stenosis or regurgitation; the aortic valve is most frequently affected, followed by the mitral valve [2]. VHD can also be classified as primary or degenerative (where the cause lies in the valve's structure, such as ageing) or secondary or functional (resulting from non-physiological cardiac remodelling, as occurs, for example, following a myocardial infarction) [3].

Epidemiological data on VHD is likely underestimated, as access to the diagnostic imaging and testing required for confirmation is not always available in less industrialised countries [3]. In 2017, aortic stenosis (AS) accounted for 61% of deaths, while 15% were attributed to mitral valve disease (regurgitation) (global-level data) [4]. In 2019, the estimated number of patients with VHD was approximately 41 million (including rheumatic heart disease (RHD) in poorer nations), with economically advanced countries recording cases of degenerative VHD (affecting the aortic and mitral valves) and infectious VHD (non-rheumatic endocarditis) [5,6]. These valvular conditions account for approximately 80% of deaths in highly industrialised countries; specifically, the predominant conditions are aortic valve calcification and mitral regurgitation (MR), while tricuspid valve disease is less common [1].

In developing countries, RHD, resulting from Group A streptococcal infection, is the leading cause of VHD. RHD affects more than 70% of countries and 0.49% of the global population (2015 data) - including India, China, Pakistan, and sub-Saharan nations - and specifically affects the mitral and aortic valves, causing stenosis [7,8]. RHD presents at a young age, whereas mortality rates are high among the elderly [7]. Due to migration towards economically developed nations, the incidence of RHD could rise again [3].

Risk factors for non-rheumatic VHD have been identified (2019 data), including high blood pressure (particularly in males and individuals over 55), high salt intake, and lead exposure [1]. Genetic abnormalities - such as bicuspid aortic valves or mitral valve prolapse - can cause VHD [9]. The ageing of the population contributes to the prevalence of VHD (aortic valve calcification and mitral regurgitation); smoking, diabetes, obesity, hyperlipidaemia, and air pollution also promote its onset, although the underlying mechanisms remain unclear [4,6]. Predisposing factors for infective endocarditis - and the resulting valvular disease - are on the rise. The use of cardiac devices (pacemakers, defibrillators, and biological or mechanical valves), illicit drug use, the widespread use of antibiotics (which foster environments conducive to the growth of bacteria such as *Streptococcus*, *Staphylococcus*, and *Enterococcus*), and cancer therapies all increase the risk of endocarditis and VHD [6].

This article briefly reviews the classifications and significance of VHDs, as well as the diagnostic tests used, to provide the reader with a clear overview. It highlights key information on pre-rehabilitation management and physical activity recommendations for the post-intervention period (following surgical or transcatheter valve procedures), in line with current guidelines. The aim of this narrative review is to underscore the importance of inspiratory muscle training (IMT) for patients undergoing valve replacement and/or repair; it identifies gaps in the literature and proposes hypotheses to address them, aligning with the perspective of the Italian Association for Cardiovascular Rehabilitation (AIRC). Finally, the article emphasises the need for further research into rehabilitation (CR (cardiovascular rehabilitation) and IMT) for patients with VHD.

## Review

Selection criteria

We conducted a comprehensive literature search in the PubMed database. The research covered texts from 2020 to 2026 (August). Studies (written in English, meta-analyses, randomised controlled trials, cohort studies, observational studies, narrative reviews) were selected based on the MeSH terms: valvular heart disease, aortic valve stenosis and regurgitation, mitral valve stenosis and regurgitation, tricuspid valve stenosis and regurgitation, pulmonary valve stenosis and regurgitation, exercise and cardiac rehabilitation. The exclusion criteria were as follows: rehabilitation research involving animal models.

Several older studies, dating back to 1991-2017, are cited. The reason for this is that these studies do not necessarily focus on rehabilitation but rather on physiological concepts.

The text is a narrative review, and the data highlighted do not include any assessment of the strengths or weaknesses of research in the field of rehabilitation, nor do they include other details such as sample sizes or risk of bias. We note a lack of concrete data on rehabilitation for patients with VHD. We used PubMed as our database because it is the largest and most important platform for scientific research worldwide.

Aortic valve disease: stenosis

AS has the highest prevalence in economically developed countries, with causes categorised as degenerative/calcific (81.9%), rheumatic (11.2%), genetic (5.6%), and endocarditic (1.3%) [6]. The prevalence of AS among elderly patients (over 75 years of age) is approximately 40%, with pathological progression of the valve structure affecting cardiac haemodynamics at about 2% per year [6]. In North America and Europe, AS affects 962.3 million people over the age of 60, with projections suggesting that by 2050, 2.08 billion individuals could be affected [10]. AS affects approximately 0.2% of people aged 50-59, 1.3% of those aged 60-69, 3.9% of those aged 70-79, and about 9.8% of those aged 80-89 [4]. The risk of developing calcific AS increases by a factor of 1.7 to 2.5 for each decade of age [11]. Data from 2019 indicate that AS was the cause of death for 127,000 people worldwide [4].

One cause of functional and structural valvular alteration is linked to cancer therapy involving mediastinal radiation and chemotherapy (with higher rates observed in lymphoma and breast cancer), which cause the aortic and mitral valves to thicken and calcify (with a latency period of 10-20 years); a less frequent cause is transthyretin amyloidosis [6,11]. Another less common cause is the presence of a bicuspid valve - found in 1-2% of the population - which is often associated with Turner syndrome or other rare genetic disorders (Loeys-Dietz syndrome, thoracic aortic aneurysm and dissection (TAAD) syndrome, Andersen syndrome, Shone syndrome, and velocardiofacial syndrome) [4,9]. The presence of a single valve cusp or multiple cusps is extremely rare [2,12].

The aortic valve area is approximately 3 cm², but in the presence of symptomatic stenosis, it decreases to <1 cm². The most common symptoms are angina, exertional dyspnoea, and potential syncope, with subsequent development of heart failure [4]. The risk of sudden death in severe but asymptomatic AS is estimated at approximately 1% per year, whereas in symptomatic patients it is estimated at approximately 10-20% per year [10].

There are no pharmacological therapies that can prevent or slow the progression of stenosis. Appropriate approaches include surgery or transcatheter intervention (TAVR), particularly when the patient is symptomatic and the ejection fraction falls below 50% [4]. If surgery or TAVR is not feasible, mortality reaches 94% within five years of diagnosis [4]. Calcific stenosis is progressive and, like all chronic diseases, affects the entire body system; AS is associated with endothelial dysfunction, atherosclerosis, and chronic systemic inflammation [11]. The onset of symptoms in AS is delayed if the coronary arteries are free of lesions [11].

In the presence of risk factors such as hypertension, dyslipidaemia, obesity, diabetes, and smoking, chronic kidney disease is another potential trigger for calcium accumulation on the aortic valve; the co-occurrence of these chronic conditions further increases the prevalence of calcific AS [11]. It can be inferred that lifestyle changes - such as not smoking (and avoiding secondhand smoke) and moderating food and alcohol intake - could help prevent calcific AS, though further large-scale data are awaited. Through mechanisms not yet fully elucidated, low-income individuals and White patients have a higher likelihood of developing calcific AS (Figure 1) [11].

**Figure 1:**
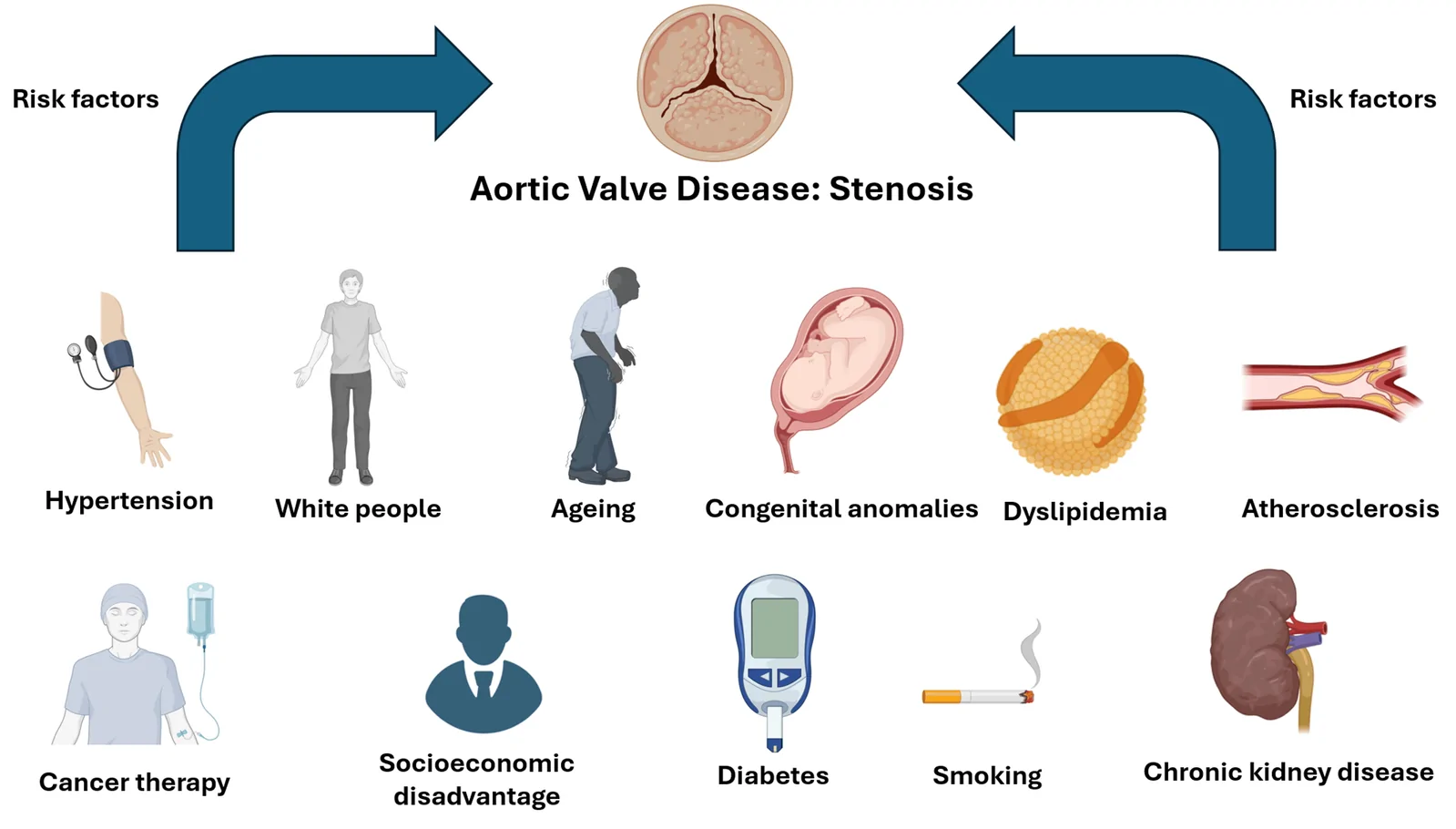
Aortic valve disease: stenosis The image schematically represents potential risk factors for aortic valve stenosis, according to current literature. Risk factors: dyslipidaemia, atherosclerosis, ageing, smoking, cancer therapy, socioeconomic disadvantage, chronic kidney disease, hypertension, diabetes, congenital anomalies, White people. Image credit: Created by Bordoni B. using BioRender.com.

Aortic valve disease: regurgitation

Aortic valve regurgitation (AR) is the third most common cause of VHD in industrialised countries, after AS and mitral valve regurgitation [3,6]. Causes include bicuspid or quadricuspid valve formation, RHD, endocarditis, connective tissue disorders, ageing, calcification, Crohn's disease, Whipple's disease, genetic conditions (Marfan syndrome, Turner syndrome, osteogenesis imperfecta), and non-physiological adaptation to aortic root dilation caused by diastolic hypertension [2-4,12]. Symptom progression is typically slow (dyspnoea, angina, syncope) but can be sudden (cardiogenic shock) following events such as trauma, aortic dissection, surgical complications, myxomatous valve degeneration, aortitis and arteritis, or acute endocarditis [6]. Risk factors associated with progression of AR include male gender, young age at diagnosis, and native valve abnormalities [4,6,7].

The prevalence of AR is not well established. In the United Kingdom, the prevalence is approximately 1.6% (2016 data) among individuals over the age of 65, whereas an American study (1999 data) reported a prevalence of about 4.9% in a population with a mean age of 28 years [6,7]. A larger study also found that AR affects 4.9% of the Western population, with a mean age of 28 years [7]. In China, the prevalence of AR is approximately 1.1% among those over 60 years of age; in Sweden, it is approximately 1.8% among those over 65 years of age [4].

With a bicuspid valve, females appear to be at higher risk of AR than males [6]. Currently, no pharmacological therapies can improve clinical condition; for severe AR (associated with decreased ejection fraction), the indicated approach is surgical or transcatheter intervention [6,7,13].

Mitral valve disease: regurgitation

MR affects approximately 24.9 million people in the Western world (2019 data), with 34,171 deaths recorded among elderly patients in 2019 [3,14]. In highly industrialised countries, primary MR is primarily associated with myxomatous degeneration (affecting the valves and related structures) - or Barlow's disease - and is more common in younger individuals; functional MR is more closely linked to advanced age and is associated with morphological and functional alterations of the left ventricle and/or left atrium, while the valve structure itself remains preserved [3,6]. MR due to valvular calcification is very common in the elderly population [7].

MR is the second most common VHD; its most well-known causes include valvular prolapse (22%), RHD (16%), myocardial infarction (30%), and dilated cardiomyopathy (26%) (2006 data) [6]. In industrialised countries, the prevalence of mitral valve prolapse is approximately 2-3%, with a higher incidence in women [9]. Prolapse involves alterations or ruptures of the papillary muscles or chordae tendineae, as well as defects in the valve leaflets and/or the annulus; there appear to be no differences across ethnic groups [9]. Prolapse can be associated with sudden death, with an annual rate of 1%; however, this figure is higher when patient age is considered, as young individuals may experience sudden death at a rate of 4-7% [9]. The symptoms of MR are typical of cardiac disease - such as angina, dyspnoea, exercise intolerance (albeit less severe than in AS), and lower-limb oedema [7,9]. MR often presents with atrial fibrillation and reduced systolic function [7].

Congenital conditions associated with mitral valve prolapse include Marfan syndrome, MASS (mitral valve, aorta, skeleton, and skin) phenotype, Loeys-Dietz syndrome, Fragile X syndrome, aneuploidy, Ehlers-Danlos syndrome, polycystic kidney disease, osteogenesis imperfecta, Ebstein's anomaly, juvenile polyposis syndrome, and Williams-Beuren syndrome [9]. Other, less common causes include oncological radiation therapy and endocarditis [2].

Currently, there are no pharmacological therapies that improve the clinical picture; in cases of severe MR - as with valvular diseases in general (characterised by reduced ejection fraction and persistent symptoms) - the indicated approach is surgical or transcatheter intervention, in accordance with American (American College of Cardiology/American Heart Association, ACC/AHA) and European (European Society of Cardiology/European Association for Cardio-Thoracic Surgery, ESC/EACTS) guidelines [13].

Mitral valve disease: stenosis

The predominant cause of mitral stenosis (MS) is RHD. In countries where RHD is prevalent, approximately 30 million patients (predominantly young) are affected, one-third of whom present with MS [7]. In developed countries, MS accounts for only 0.1-0.2% of cases, with annular calcification the primary cause - particularly among the elderly and patients undergoing dialysis [2,7]. Other rare causes of MS include mucopolysaccharidosis, Fabry disease, systemic lupus erythematosus, and congenital abnormalities (such as a parachute mitral valve and supramitral valve) [7].

Symptoms of MS are not present immediately. Severe symptoms occur when the valve orifice area is less than 1 cm² (normal area: 4-6 cm²). These include exertional and resting (supine) dyspnoea, lower-limb oedema (due to right-sided heart failure), haemoptysis (due to pulmonary hypertension), and hoarseness and dysphagia resulting from compression of the vagus nerve and oesophagus by the hypertrophied left ventricle [4].

Depending on the clinical presentation and the surgeon's final decision, the therapeutic approach may involve surgery or transcatheter mitral valve replacement [13,15].

Tricuspid valve disease: regurgitation

Tricuspid regurgitation (TR) is a less common form of valvular disease, yet it is associated with a high post-diagnosis mortality rate - exceeding 42% after three years [4,6]. Comprehensive epidemiological data on TR are lacking. A 2016 study reported that 2.7% of the elderly population in England has TR, whereas a 2019 study found a prevalence of 0.55% in the US population [4]. In the Chinese population, the prevalence of TR ranges from 0.8% to 1.1% [3,7]. Females and individuals over the age of 75 (with a prevalence of 4%) appear to be most affected [6,7]. TR primarily manifests as a morphological and functional abnormality secondary to cardiac conditions (95%) - specifically left-sided heart disease (54.4%), atrial dysfunction (24.3%), and pulmonary disease (17%) [6].

The remaining proportion of cases (primary disease) stems from disruption of tricuspid valve integrity, such as that caused by endocarditis or congenital conditions/defects (e.g., Ebstein’s anomaly) [6,9]. Symptoms associated with severe TR (and reduced ejection fraction) are linked to right-sided heart failure - including weight gain, jugular venous distension during inspiration (Kussmaul’s sign), hepatojugular reflux, ascites, fatigue and exercise intolerance, atrial fibrillation, dyspnoea, dyspepsia, diarrhoea, cachexia, and potential cardiogenic shock [16]. When TR becomes severe and presents a dangerous haemodynamic profile, management strategies involve surgery or transcatheter interventions [6,13].

Tricuspid valve disease: stenosis

Tricuspid valve stenosis (TS) is an uncommon condition; in 90% of cases, it is caused by rheumatic disease, though other causes include lupus erythematosus, carcinoid syndrome, antiphospholipid antibody syndrome, hypereosinophilic syndrome, metastases, and congenital conditions (pulmonary atresia, Ebstein's anomaly, Whipple's disease, and Fabry disease) [9,17,18]. Other causes stem from non-physiological iatrogenic surgical changes - such as the placement of a device within the subvalvular region - or represent late complications of valve replacement [17,18]. Additionally, certain medications, such as anorectics or specific psychedelic drugs, can induce TS [17]. Severe TS presents typical symptoms of systemic cardiovascular congestion, although the prognosis is relatively favourable [17]. The management of severe TS invariably involves surgical or transcatheter intervention [17].

Multivalvular heart disease (MVHD)

MVHD is defined by functional abnormalities (stenosis and/or regurgitation) in two or more heart valves [6,7]. MVHD affects 10-20% of patients with VHD; these patients tend to be older, include a higher proportion of non-Hispanic Black individuals, and frequently present with atrial fibrillation, chronic kidney disease, and rheumatic disease [19]. Rheumatic disease appears to be the primary cause of this condition, with a higher incidence among women [6,7]. Compared with patients with single-valve dysfunction, those with MVHD have a lower ejection fraction and greater symptom severity; however, they have a lower prevalence of hyperlipidaemia and a lower body mass index and a higher incidence of heart failure [6,19].

The most common form of bivalvular dysfunction in patients with MVHD involves the mitral and tricuspid valves (MR/TR), followed by the aortic and tricuspid valves (AS/TR) and the aortic and mitral valves (AS/MR, or, to a lesser extent, AR/MS) [19]. Compared with patients with single-valve disease, those with multiple valvular pathologies have a less favourable prognosis; furthermore, the choice between surgical and transcatheter interventions depends heavily on the morphological adaptation of the cardiac chambers, as dysfunction in one valve can impair the function of another, thereby accelerating the progression of the clinical condition (Figure 2) [7,19-21].

**Figure 2:**
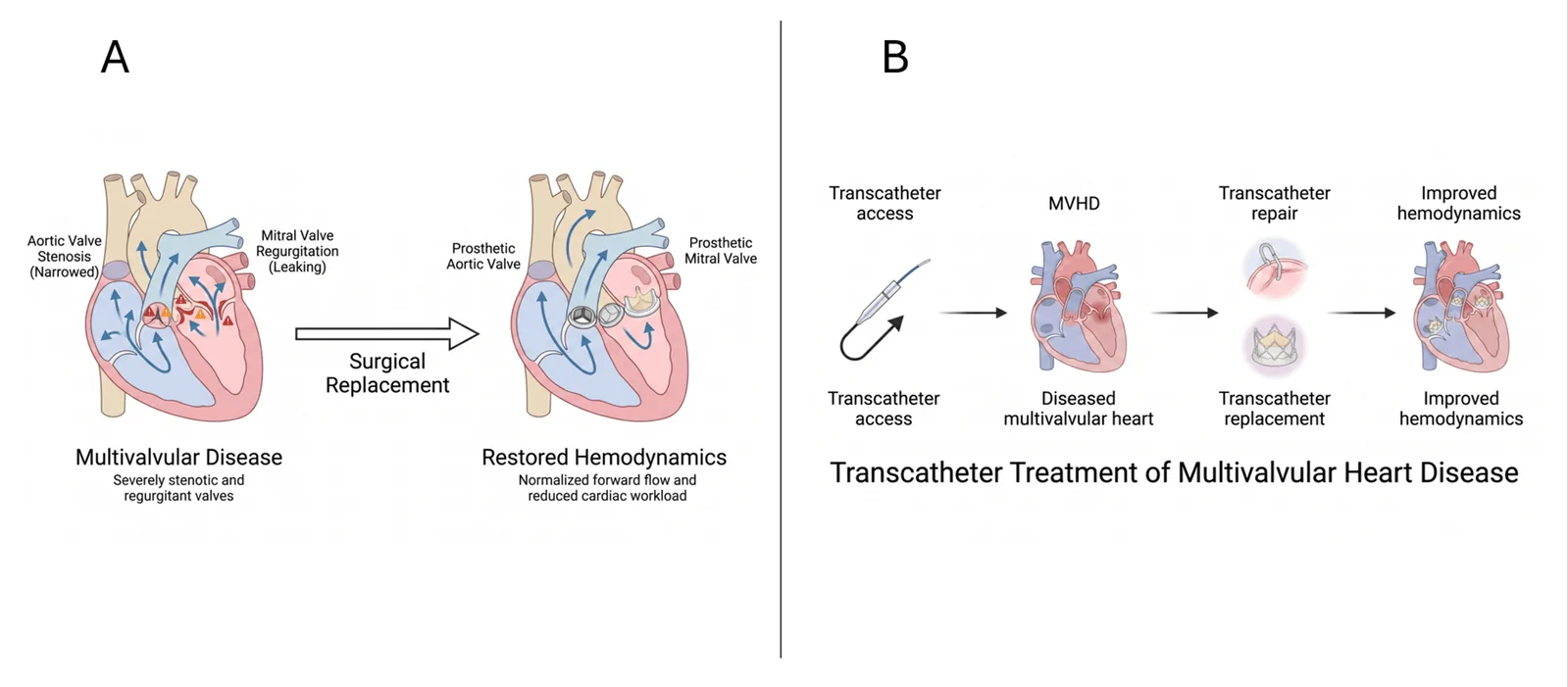
Surgical and transcatheter approaches Image A schematically illustrates the surgical approach to aortic valve replacement for stenosis (AS) and to mitral valve replacement for regurgitation (MR); Image B shows heart valve repair and/or replacement using a transcatheter approach, also in the setting of multivalvular heart disease (MVHD). Image credit: Created by Bordoni B. using BioRender.com.

There are no guidelines for cases of MVHD and concomitant coronary artery disease (CAD), a scenario in which the therapeutic choice must always be individualised [13]. A 2019 study found that approximately one-third of patients with VHD have concomitant CAD; the coexistence of CAD and VHD increases the complexity of the clinical approach [22]. There is currently no consensus on which interventional approach - surgery or transcatheter - is safer for the patient [23].

Generally, an interventional approach aims to replace the malfunctioning valve, with the choice between a mechanical valve (55% of cases) and a biological valve (45% of cases) based on the patient's clinical status and preferences [24]. Mechanical valves require more long-term management because of the need for anticoagulants, whereas biological valves (porcine or bovine pericardium) require significantly less anticoagulant management [24].

Recovery time with transcatheter procedures (using balloon-expandable or self-expanding valves) is shorter than with sternal or minimally invasive surgery [24].

Pulmonary valve disease: stenosis and regurgitation

Pulmonary valve stenosis (PS) is usually congenital (associated with conditions such as tetralogy of Fallot, univentricular heart, Noonan syndrome, or Ebstein anomaly) and is very rare when present in isolation. PS affects approximately 8% of the population with congenital heart defects, with a higher prevalence in Asian countries than in Western countries [25]. Treatment is selected based on the severity of the stenosis (peak gradient ≥ 64 mmHg) and/or the presence of symptoms, such as cyanosis, heart failure, and exercise intolerance [25]. Secondary causes may include advanced age (calcification), rheumatic disease, neoplastic lesions, or post-surgical cardiac complications [25]. PS causes pulmonary hypertension, dysfunction, and ischaemia of the right heart chambers, and arrhythmias. In cases of severe stenosis, the approach involves repair or replacement [25].

One complication of the interventional approach is pulmonary regurgitation (PR), observed in approximately 70% of patients; PR increases mortality [26]. Only about 10% of patients present with idiopathic rather than iatrogenic PR [26]. For severe PS, the recommended approach is catheter-based balloon valvotomy, whereas for PR, the recommended approach is to use a pericardial or synthetic polymer patch [27].

Instrumental examinations

Various instrumental tests are available to accurately identify VHD/MVHD, with the choice depending on equipment availability. Echocardiography is the initial step in identifying a lesion; specifically, transoesophageal echocardiography (TEE) or 3D echocardiography provides superior imaging compared with standard 2D ultrasound [6,7,27]. Clinicians may opt for dobutamine stress echocardiography to better correlate symptoms with valvular status [7]. Further options for a more accurate diagnosis include 3D intracardiac echocardiography, cardiac computed tomography (CCT), and cardiac magnetic resonance imaging (CMR); CCT or CMR are typically selected for pre-procedural assessment [6,7,27].

Conservative management

The ACC/AHA and ESC/EACTS guidelines call for the management of patients with VHD by a specialised team; the former advocate referring patients to centres specialising in valve care, whereas the latter recommend adhering to the specific procedures of individual hospital centres [13]. A common feature of these guidelines is the multidisciplinary involvement of various healthcare professionals, though neither specifies how to organise pre-rehabilitation. Before resorting to invasive intervention and/or hospitalisation, continuous surveillance via clinical examinations is recommended to monitor disease progression [13]. There are no specific guidelines beyond the use of medications to manage comorbidities [10]. Patients with non-severe VHD and stable clinical status may engage in physical activity tailored to their physical and social characteristics [14,28]. Moderate physical activity includes endurance training (ET), resistance training (RT), and IMT [14,28]. Moderate physical activity helps reduce post-cardiac surgery and pulmonary complications and shortens hospital length of stay [29,30,31]. In particular, pre-interventional IMT for patients awaiting TAVR has been shown to reduce the rate of post-surgical pneumonia by 75% and shorten the length of hospital stay by 25% [32].

European data indicate that patients awaiting aortic valve replacement face a waiting period of 7-14 months; among these patients, 13% require unplanned hospitalisation due to disease progression and severity, and 6% die before admission [33]. US data show an average wait of approximately three months, with a mortality rate of 5.2% among these patients [33]. This waiting period leads to deterioration in postoperative clinical status [33].

Pre-rehabilitation is particularly beneficial for frail patients, as it can improve functional capacity, quality of life, and frailty indices [34]. There is a lack of data and research on pre-rehabilitation, as well as a lack of established practice for initiating rehabilitation pathways prior to hospitalisation or for maintaining a non-severe clinical condition-a gap highlighted by the absence of recommendations in the latest 2026 ESC guidelines [35].

Post-valve surgery rehabilitation

According to the 2026 ESC guidelines, cardiovascular rehabilitation (CR) - centred on physical activity - offers benefits that cannot be matched by pharmacological therapy [35]. A 2021 Cochrane meta-analysis found no improvements in mortality, quality of life, or hospitalisation duration among 580 patients who underwent surgery or percutaneous interventions [36]. No adverse effects were reported in this patient population undergoing CR [5,10,32]. Two articles (a meta-analysis and a narrative review from 2022 and 2023) reported improvements in certain functional parameters - including peak VO2, 6MWT results, and quality of life - although the rehabilitation protocols were not comparable and the reported details were incomplete [8,32].

A more recent meta-analysis (685 patients undergoing transcatheter aortic valve implantation, TAVI) evaluated functional adaptations during CR lasting two weeks to two months, depending on the protocol used (which varied across studies). CR improved 6MWT performance and various parameters of cognitive function and depression, alongside an increase in daily motor independence (Barthel Index) [10].

Two trials involving patients undergoing surgical valve treatment evaluated functional outcomes with follow-up of 3-12 months. The trials (involving TAVR patients) utilised CR featuring moderate-intensity continuous training (MICT) three times a week; improvements were observed in pulmonary functional parameters, such as peak VO2, and motor functional parameters, such as the 6-minute walk test (6MWT), alongside a reduction in low-density lipoprotein cholesterol [37,38]. Another 2024 meta-analysis (685 TAVI patients) - covering CR programmes lasting between two weeks and two months, with training variables that varied across studies - highlighted improvements in motor function (6MWT), cognitive parameters, and depression levels related to daily motor autonomy [39].

Post-surgical CR for valvular disease is beneficial - regardless of patient frailty, sex, or the timing of rehabilitation - because most patients undergoing surgery already present with cardiac damage, often because valvular issues remain asymptomatic until the initial cardiology consultation [13,40]. Furthermore, patients undergoing valvular surgery appear to have lower muscle performance capacity than those undergoing coronary artery bypass grafting (CABG) [14]. Another reason to implement CR in this patient population is the presence of comorbidities. Many patients present for surgery with multiple comorbidities and sarcopenia, resulting in greater frailty and longer hospital stays [41,42]. CR could accelerate functional recovery following cardiac surgery in frail and sarcopenic patients [43].

Based on the limited available information, the benefits of CR after mitral valve surgery remain inconclusive; most trials have focused on patients with aortic conditions or who have undergone aortic surgery [14]. Patients with mitral valve disease likely have lower exercise tolerance than those with aortic disease [14]. The reasons for this are unknown.

The literature reveals a scarcity of data on CR and VHD, as well as inconsistencies in operational protocols or a lack of detail on physical activity [10,44-46]. In my view, this latter issue stems from cardiologists who organise research studies or meta-analyses not supervising patients during physical activity but instead relying solely on clinical data; there is no equal partnership with the healthcare professionals who deliver and organise the rehabilitation training programme. Furthermore, the optimal duration of a CR programme following valvular surgery remains unclear. Some authors advocate a three-month course - similar to that for patients undergoing CABG - while others consider determining the most appropriate CR duration for this patient population to be less critical [47-50].

Post-valvular surgery rehabilitation: IMT

Another approach within CR is IMT, which is often underestimated or not considered an integral part of physical activity [36,49,50]. TAVI patients undergoing post-ICU IMT show improvements in clinical parameters, including shorter hospital stays and increased ventilatory capacity and inspiratory muscle strength [32,48].

Why is IMT important for patients with valvular disease undergoing surgical procedures? Patients undergoing TAVI experience autonomic imbalances - characterised by heightened sympathetic activity relative to parasympathetic activity - alongside disturbances in heart rhythm and blood pressure [10]. Optimal diaphragmatic function, supported by IMT, improves autonomic responses, thereby benefiting blood pressure and heart rhythm [10,51,52]. Furthermore, combining IMT with CR in TAVI patients reduces hospital stays, improves functional exercise capacity (as measured by the 6MWT), and accelerates recovery of pulmonary function and inspiratory muscle strength [53]. In the latter study, the maximal inspiratory pressure (MIP) - measured by spirometry - was used (as is standard practice) to set the IMT device resistance (30-60%); the training regimen consisted of five minutes of IMT daily for 3-5 days during the hospital stay [53]. However, specific details are lacking on how IMT was integrated into the daily training programme (e.g., alongside stationary cycling), the rationale for selecting those resistance levels, and the duration of the pause between successive inhalations.

Patients undergoing cardiac surgery (median sternotomy or minimally invasive procedures) for valve replacement or repair (aortic, mitral, and tricuspid) are susceptible to phrenic nerve injury (often transient), resulting in more pronounced impairment of pulmonary function and increased mortality and morbidity [53-55]. Without a CR or IMT programme, pulmonary function does not improve one-year post-surgery, with MIP values lower than pre-surgical levels [54]. A reduction in MIP is associated with a sixfold increase in the 90-day mortality rate following valve surgery [54]. Furthermore, MIP correlates with all measures of muscle function and performance (such as the 6MWT and the Berg Balance Scale) [54,56]. IMT is a fundamental component of rehabilitation after an invasive procedure. There are currently no precise, standardised guidelines for the use of IMT; resistance levels range from 30% of MIP (in TAVI patients) to 80% of MIP (in patients after surgical mitral valve replacement) [53,56]. The reasons for this variability remain unexplained, as do variations in training parameters (duration, rest intervals, integration with other physical activities, etc.) [53,56].

How long might phrenic nerve or diaphragmatic dysfunction persist after cardiac valve surgery, given factors such as sarcopenia, comorbidities, patient frailty, and the specific type of valve procedure? There is no precise data that accounts for all these variables. Generally, phrenic nerve injury and diaphragmatic dysfunction are common; findings range from frank elevation of a hemidiaphragm (27% of cases) to reduced mobility (70.8%) and decreased diaphragmatic thickness (75%) in the postoperative period [57-59]. It should be noted that elevation of a hemidiaphragm does not necessarily indicate phrenic nerve damage, as it may also result from pleural effusion or atelectasis [60]. Diaphragmatic recovery times can range from a minimum of one month to 12 months - or even 18 months in cases of partial phrenic nerve injury [61]. Indeed, pulmonary complications resulting from post-cardiac surgery diaphragmatic dysfunction can persist for more than a year after the procedure [62].

Position statement of the Italian Association for Cardiovascular Rehabilitation: IMT

Training designed to increase peak VO₂ must last at least 2-4 weeks; otherwise, improvement cannot be demonstrated within the context of CR [63]. Furthermore, as noted in the previous section, the minimum timeframe for supporting diaphragmatic recovery after cardiac surgery is one month [61]. IMT must be performed for at least one month post-surgery to yield benefits.

Any CR programme must be based on specific guidelines: FITT-VP (frequency, intensity, time, type, volume, and progression) [35,48]. This allows CR to be tailored to the patient's specific needs and capabilities, following the assessment-training-reassessment (ATR) principle [48].

The diaphragm muscle is composed of approximately 55% aerobic (red) fibres, with the remainder consisting of anaerobic (white) fibres (Type IIA: ~21%; Type IIX: ~24%) [64]. Red fibres are activated during eupneic breathing or endurance physical activity, whereas white fibres are recruited during more intense or sudden actions, such as coughing [65,66]. Standard physical activity - such as cycling, treadmill exercise, or weightlifting - does not produce equivalent adaptations in the limbs and the diaphragm; instead, IMT is required to provide more comprehensive diaphragmatic stimulation, utilising inspiratory resistance levels capable of engaging both white and red fibres [67]. The resistance level used to ensure effective IMT (determined from MIP measured by spirometry) should not exceed 50% of the subject's maximum inspiratory mouth pressure (PImax). Exceeding this threshold risks premature diaphragmatic fatigue, leading to increased reliance on accessory inspiratory muscles and requiring a diaphragmatic recovery period of approximately 30 minutes [52,68]. Furthermore, the diaphragm exhibits reduced contractile activity in post-surgical patients [69].

For effective IMT, inspiratory resistance levels must be set to respect the patient's subjective PImax without exceeding the 50% threshold, while adhering to parameters that specifically target both red and white muscle fibres. Furthermore, it is necessary to determine how to integrate IMT into other CR activities and to schedule sessions within the rehabilitation cycle that avoid consistently high loads, thereby preventing Overtraining Syndrome (OTS) [70]. The concept of progression does not imply a constant increase in training effort; indeed, even athletes - to avoid OTS - incorporate training sessions with low workloads, or periods of "deloading" (for resistance training) or "tapering" (for endurance training) [71,72]. There is a lack of data in the literature regarding this approach to training management in patients. This approach might be referred to as FITT-VP-R (rest).

It is necessary to separate the two metabolic training modalities (endurance and resistance) within CR-IMT, as performing them consecutively could delay peripheral adaptation in one component relative to the other (aerobic or anaerobic). If high-intensity aerobic exercise precedes resistance training, parameters of strength and contractile capacity are reduced. Similarly, if resistance training intensity is high, aerobic adaptation is compromised [73,74]. However, findings across the literature are not entirely consistent [75]. Data on patients in the literature are lacking. Notably, patients with chronic conditions and comorbidities exhibit myopathic adaptations characteristic of the pathology. For instance, patients undergoing valve surgery who also have chronic obstructive pulmonary disease (COPD) or chronic heart failure (CHF) experience a reduction in red fibres in peripheral muscles and an increase in red fibres in the diaphragm - changes occurring within a pro-inflammatory metabolic environment. Such phenotypic disparities do not represent a physiological adaptation but rather contribute to performance decline and dyspnoea [76,77]. Deconditioned musculature situated in a chronic inflammatory environment will require a longer recovery period [78].

The response to endurance training resembles that induced by injury, with an inflammatory peak at 24 hours; the inflammation triggered by the training stimulus facilitates supercompensation or tissue regeneration [78]. A mismatch between the muscle's immune response capacity and the training stimulus - combined with inadequate rest between sessions - can lead to muscle damage and subsequent fibrosis [78]. Elderly patients commonly exhibit chronic, subclinical muscle inflammation, myopathy, and fibrosis, characterised by elevated levels of pro-inflammatory "gerokines" (a group of signalling molecules, including cytokines, chemokines, and proteases, or the ageing secretome) [78,79]. Patients with chronic cardiac conditions present a heightened systemic inflammatory environment [80].

It can be inferred that elderly surgical patients undergoing valve repair or replacement with chronic comorbidities will exhibit reduced adaptability of the peripheral and diaphragmatic muscles. In such patients - characterised by an untrained muscular state, chronic pathologies, and advanced age - a light training load will most likely elicit a far more intense metabolic and inflammatory response, necessitating longer recovery times than those of a peer of the same age and sex. The greater the exertion to which the patient is subjected, the longer the time required for cellular regeneration [81]. Incorporating low-intensity training sessions - where the patient remains active but avoids reaching the point of fatigue (deload/taper) - into the weekly routine could prove to be a useful strategy, though further data and studies are required.

How should one determine the number of sets, repetitions, and rest intervals between sets to optimise diaphragmatic stimulation? As discussed in detail in a previous article, one can follow the guidelines of the American College of Sports Medicine (ACSM) [52,82]. While there is no definitive evidence on the best time of day - morning or afternoon - to schedule aerobic and anaerobic training, maintaining a consistent routine helps achieve benefits more quickly (Table 1) [83].

**Table 1:** Parameters for diaphragmatic stimulation via inspiratory muscle training (IMT)

Inspiratory muscle split training for red fibres - afternoon	Inspiratory muscle split training for white fibres - morning
30% of maximal inspiratory pressures	50% of maximal inspiratory pressures
2-3 sets of 15 inhalations	2-3 sets of 8-12 inhalations
90-second rest between sets	3-minute rest between sets
2-3 days during the week	2-3 days during the week

For patients with VHD who have undergone repair or replacement - whether via thoracic surgery or catheter-based procedures - AIRC recommends IMT using the parameters published in Cureus and shown in Table 1 [52]. In this article, we further suggest incorporating a one-week "deload" or taper phase midway through the CR to protect patients against inadequate recovery between training sessions. Our recommendations are based on existing literature on training and diaphragm; they are not mandatory but rather suggestions that warrant further investigation through experimental studies. The ideal duration for a CR programme incorporating IMT is three months, although we acknowledge that delivering rehabilitation outside the cardiology department is not always feasible [84]. Finally, the AIRC committee suggests targeting "red fibres" with IMT before endurance training and "white fibres" before anaerobic training, thereby optimising the respiratory muscles' use of energy resources.

## Conclusions

VHD is a major cause of cardiovascular morbidity and mortality worldwide. This narrative review has highlighted the importance of IMT for patients undergoing valve replacement and/or repair, identified gaps in the literature, and proposed hypotheses to address them from the perspective of the AIRC. Further experimental studies are needed to better define the rehabilitation needs of patients with VHD.
